# Immunogenicity and efficacy of a *Mycoplasma hyopneumoniae*/porcine circovirus type 2 combination vaccine against experimental *Mycoplasma hyopneumoniae* infection in pigs

**DOI:** 10.1186/s13567-026-01808-9

**Published:** 2026-07-07

**Authors:** Li Wang, Filip Van Immerseel, Ilias Chantziaras, Karina Sonalio, Ana Karolina Panneitz, Xiaoyan Ma, Roman Krejci, Dominiek Maes, Bert Devriendt

**Affiliations:** 1 Department of Internal Medicine, Reproduction and Population Medicine, Faculty of Veterinary Medicine, Ghent University, Salisburylaan 133, 9820 Merelbeke, Belgium; 2 Department of Pathobiology, Pharmacology and Zoological Medicine, Faculty of Veterinary Medicine, Ghent University, Salisburylaan 133, 9820 Merelbeke, Belgium; 3 CEVA Santé Animale, Libourne, France; 4 Laboratory of Immunology, Faculty of Veterinary Medicine, Ghent University, Salisburylaan 133, 9820 Merelbeke, Belgium

**Keywords:** *Mycoplasma hyopneumoniae*, combination vaccine, experimental study, humoral immunity, cell-mediated immunity

## Abstract

Vaccination is an important control measure against infections with *Mycoplasma hyopneumoniae* (*M. hyopneumoniae*), the primary pathogen of enzootic pneumonia in pigs. The present study investigated local and systemic humoral and cell-mediated immune responses and efficacy upon vaccination of piglets with a combined vaccine containing inactivated *M. hyopneumoniae* and porcine circovirus 2d (Cirbloc^®^ M Hyo, Ceva), followed by experimental *M. hyopneumoniae* infection (D21). Forty-five piglets were allocated to three groups, namely the not-vaccinated and infected (C) (*n* = 20), vaccinated and infected (V) (*n* = 20), and not-vaccinated and not-infected (NC) groups (*n* = 5). Vaccination was applied once (D0) intramuscularly. Four pigs from the V and C groups were euthanized 2 weeks post-infection (D35), and the remaining pigs 2 weeks later (D49). Blood, lung tissue, bronchoalveolar lavage fluid (BALF), and bronchial lymph nodes were collected at different timepoints. Piglets in the V group exhibited seroconversion on days 21 and 35, with markedly elevated levels of specific IgA and IgG in BALF on D35. Furthermore, the V group showed activation of interferon (IFN)-γ^+^/tumor necrosis factor (TNF)-α^+^ CD4^+^CD8α^+^ T cells and T cell proliferation on day 7, with these responses remaining elevated through D21. The concentrations of interleukin (IL)-1β, TNF-α, C–X–C motif chemokine ligand 8 (CXCL-8), and IL-17A in BALF of the C group were higher on D49, correlating with higher *M. hyopneumoniae* DNA loads. Clinical signs and lung lesions were less severe in vaccinated pigs. The combined vaccine elicited robust *M. hyopneumoniae*-specific humoral and cellular responses in blood and enhanced post-infection cellular immunity in draining lymph nodes, underscoring its protective efficacy.

**Supplementary Information:**

The online version contains supplementary material available at 10.1186/s13567-026-01808-9.

## Introduction

*Mycoplasma hyopneumoniae* (*M. hyopneumoniae*) is the etiological agent of enzootic pneumonia, a chronic respiratory disease in pigs, and an important pathogen in the porcine respiratory disease complex [1]. The pathogen is widespread in the global pig industry and is responsible for significant economic losses [2]. Pigs of all ages can be infected, but most clinical signs occur in grow-finishing pigs [3]. Clinical manifestations include chronic coughing, growth retardation, and lower feed efficiency [1]. Pigs infected with *M. hyopneumoniae* are more susceptible to other respiratory infections [4] and require more antimicrobials for treatment.

Vaccination is an important tool to control *M. hyopneumoniae* infections. It is used worldwide to minimize performance losses, clinical signs, lung lesions, and treatment costs. Unfortunately, the current vaccines only provide partial protection and do not prevent colonization and transmission [5–8]. Also, the efficacy of combined vaccines against *Mycoplasma hyopneumoniae* and other pathogens has been assessed under both field and experimental conditions. Field studies have mainly assessed ready-to-use bivalent vaccines containing porcine circovirus type 2 (PCV2) and *M. hyopneumoniae*. For example, Nielsen et al. reported improved production performance in Danish pig herds following vaccination on the basis of historical comparisons. Similarly, Tzika et al. and Ham et al. demonstrated that bivalent vaccination under field conditions effectively reduced disease impact and improved herd health status [6, 7, 9]. Under experimental conditions, Krejci et al. evaluated a PCV-2d and *M. hyopneumoniae* vaccine using challenge models with different PCV-2 genotypes and showed that vaccination reduced viral DNA levels in serum and provided early and sustained protection [10]. More recently, multivalent vaccines have been investigated. Allen et al. and Suh et al. evaluated trivalent vaccines by examining pathogen burden, lung lesion severity, and safety [8, 11]. These studies demonstrated the potential of combined vaccines in controlling porcine respiratory pathogens. However, these studies with the combined vaccines did not perform comprehensive investigations into the protective humoral and cellular immunity.

Efforts to improve the efficacy of *M. hyopneumoniae* vaccines rely on elucidating the immune responses upon vaccination and subsequent infection [12]. Inactivated *M. hyopneumoniae* vaccines usually induce seroconversion in pigs, pointing to a humoral immune response [13]. However, serum antibodies are not correlated with protection, and cell-mediated immunity is considered more important for protection. Colonization of the respiratory tract by *M. hyopneumoniae* leads to the secretion of various cytokines, which subsequently drive a robust inflammatory response [14, 15]. Although these responses may be associated with protection, the massive infiltration of lymphohistiocytic cells in the lung tissue upon infection is considered as being part of the pathogenesis and involved in the pulmonary lesions [16]. Our research group previously observed that, in vaccinated animals infected with *M. hyopneumoniae*, concentrations of the pro-inflammatory cytokines interleukin (IL)−1β and IL-6 in bronchoalveolar lavage fluid (BALF) were lower than in unvaccinated infected animals, while levels of the anti-inflammatory cytokine IL-10 were higher [17]. We also showed that *M. hyopneumoniae* vaccination may mitigate inflammatory responses by reducing IL-17A expression after infection [17, 18]. Expression of pro-inflammatory cytokines correlates positively with the severity of pulmonary tissue pathology, while the upregulation of the anti-inflammatory cytokine IL-10 may suppress ongoing inflammatory responses and also facilitate the establishment of chronic infection [19]. The relationship between these cytokines and protective or inflammatory mechanisms remains as yet unclear.

T-cell-mediated immune responses are generally considered crucial for defense against *Mycoplasma* species causing local respiratory infections, such as *M. hyopneumoniae* [17, 20]. Previous studies have demonstrated that vaccination against *M. hyopneumoniae* increases the proportion of CD4^+^CD8α^+^ T cells producing tumor necrosis factor (TNF)-α and TNF-α^+^/interferon (IFN)-γ^+^ in peripheral blood. In addition, the proportion of TNF-α^+^ CD8α^+^ T cells is markedly elevated, accompanied by substantially enhanced T cell proliferation after vaccination [17, 21]. These studies primarily assessed cellular immune responses using peripheral blood samples only. While informative, this approach provides a limited view on vaccine-induced immunity. A more comprehensive evaluation of cellular immune responses at both systemic and local levels, particularly in tissues directly involved in *M. hyopneumoniae* infection is needed, as well as longitudinal cellular immune monitoring and evaluation of local respiratory immune responses [8, 22].

The present study investigated the local and systemic immune responses of a new inactivated *M. hyopneumoniae* vaccine (strain 2940) that is combined with porcine circovirus type 2 d (ORF2 capsid protein) at different time points in pigs that were subsequently experimentally infected with *M. hyopneumoniae*. The pigs were monitored throughout the study and necropsied at 2 and 4 weeks after infection.

## Materials and methods

### Animals

The study was approved by the Ethics Committee of the Faculty of Veterinary Medicine and the Faculty of Bioscience Engineering, Ghent University (EC 2024-023).

Piglets (45; 23 males and 22 females; Naima × Piétrain) were selected from a herd with a confirmed *M. hyopneumoniae*-free status. The farm had been consistently verified as *M. hyopneumoniae*-free over recent years through a combination of serological testing, absence of clinical signs, tracheal swab analysis using nested polymerase chain reaction (nPCR), and evaluation of lung lesions. Before the initiation of the study, 40 tracheobronchial swabs were obtained from pigs of varying age groups (including weaned piglets and fattening pigs) and tested for *M. hyopneumoniae* DNA using a quantitative Real-Time TaqMan PCR (qPCR) assay. All samples tested negative. Additionally, the farm was free of other major respiratory pathogens, including porcine reproductive and respiratory syndrome virus (PRRSV) and *Actinobacillus pleuropneumoniae*. Two animals died during or shortly after the challenge infection. These two piglets were excluded from the analyses performed after challenge. Three pigs (two piglets were from the V group and one piglet was from the C group) developed mild diarrhea and lameness between D7 and D16 and were treated with amoxicillin (Duphamox^®^ LA, Covetrus, Belgium) and an anti-inflammatory drug (Melovem^®^, Covetrus, Belgium) for 3–5 days.

### Experimental design

**Figure 1:**
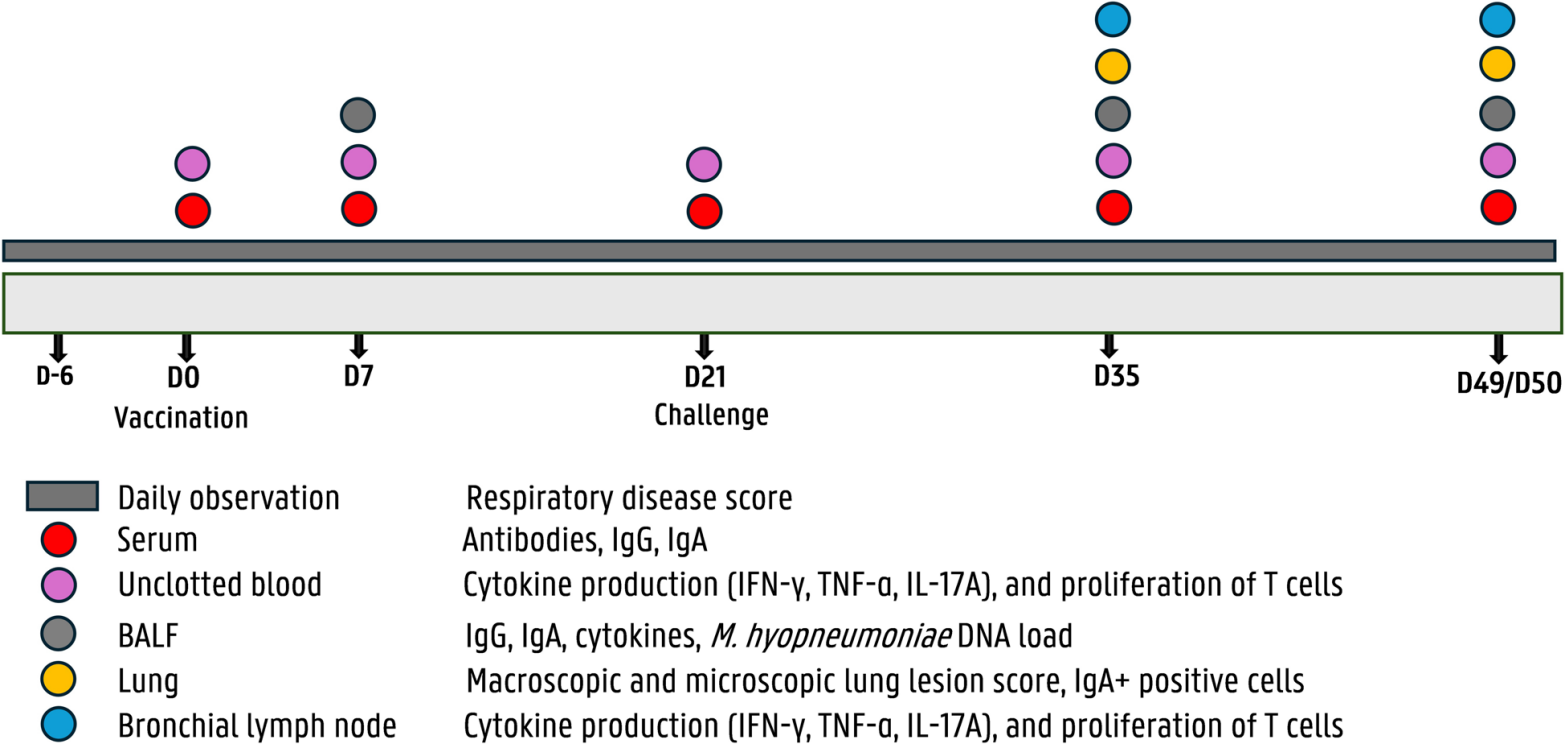
**Overview of the experimental design.** D-6: arrival of piglets in experimental facilities; D0: vaccination; D7: 1 week after vaccination; D21: experimental infection; D35: necropsy of four animals in the V and in the C group; D49: necropsy of the remaining animals. Piglets (*n* = 45) were divided into three groups. Twenty piglets were vaccinated on D0 (V) and challenged with *M. hyo* after 3 weeks (D21), 20 piglets were not vaccinated but challenged (C), the remaining 5 piglets served as the negative control (NC); Eight pigs (4 from the V and 4 from the C groups) were euthanized 2 weeks after challenge (D35), the remaining ones were euthanized at D49. Blood was collected on D0, D7, D21, D35, and D49. BALF was collected on D7, D35, and D49. Lung tissue and bronchial lymph nodes (BLN) were collected at necropsy day (D35 and D49).

The experimental design and an overview of the collected samples are presented in Figure 1. Piglets were weaned at 18 days of age and subsequently transported from the source herd to the research facilities of the Faculty of Veterinary Medicine at Ghent University. Upon arrival, the animals were randomly allocated to three experimental groups and underwent a 6-day acclimatization period. These three groups included a not-vaccinated and infected group (C) (*n* = 20), a vaccinated and infected group (V) (*n* = 20), and a not-vaccinated and not-infected (NC) group (*n* = 5). Stratified randomization was performed with consideration of body weight (to ensure comparable starting weights across groups) and sex distribution. All animals had ad libitum access to drinking water and were fed a commercial, antimicrobial-free diet.

The piglets from the C, V, and NC groups were initially housed together in five separate units (nine animals per unit) until challenge-infection (D21). From then onward, the pigs of the NC group (*n* = 5) were housed in a separate unit, while pigs from the C and V groups were mixed across the five units.

At 24 days of age (D0), piglets in the V group (*n* = 20) received an intramuscular injection of 2 mL of a combined vaccine containing inactivated *M. hyopneumoniae* and inactivated Porcine Circovirus type 2 d (Cirbloc^®^ M hyo, Ceva Animal Health). Piglets in the C (*n* = 20) and NC (*n* = 5) groups were administered 2 mL of sterile phosphate-buffered saline (PBS) via intramuscular injection. Three weeks post-vaccination, at 45 days of age (D21), piglets in the C and V groups were challenge-infected via endotracheal inoculation with 7 mL of an inoculum containing 10^7^ color-changing units (CCU)/mL of the highly virulent *M. hyopneumoniae* strain F7.2C, followed by inoculation with the low-virulent strain F1.12A the next day [18]. Piglets in the NC group received a similar inoculation protocol using 7 mL of sterile modified Friis culture medium. For all inoculations and BALF sample collection, animals were anesthetized via intramuscular injection with 0.11 mL/kg of a mixture containing zolazepam and tiletamine (Zoletil 100^®^, Virbac, Louvain-la-Neuve, Belgium) and xylazine (Xyl-M^®^ 2%, VMD, Arendonk, Belgium), corresponding to doses of 2.2 mg/kg xylazine and 4.4 mg/kg Zoletil. Four pigs of the C and four pigs of the V group were necropsied on D35. The remaining pigs were necropsied at D49.

### Sample collection

Blood samples were collected via jugular vein puncture at multiple time points: D0 (vaccination day), D7 (1 week after vaccination), D21 (challenge day), D35 (2 weeks after challenge), and D49 (4 weeks after challenge). Serum was separated and stored at −20 °C until further analysis. Additionally, non-clotted blood was collected in ethylenediaminetetraacetic acid (EDTA)-coated vacutainer tubes and used immediately to assess cell-mediated immune responses.

BALF was collected on D7, D35, and D49 using a plastic pipette attached to an automatic pipettor inserted into the trachea. The lungs were flushed with 20 mL of sterile PBS as described previously [17, 18]. At necropsy, BALF was obtained by flushing the main bronchus of the right lung lobe with 20 mL sterile PBS as described [23]. The BALF samples were stored at −80 °C until further analysis.

### Clinical and performance parameters

Throughout the study, all animals were monitored daily for a minimum of 20 min by the principal investigator during morning hours (08:00–10:00 AM). During these observations, clinical assessments were conducted. These included the evaluation of coughing severity using the Respiratory Disease Score (RDS) [24] as well as documentation of other clinical signs such as reduced appetite, diarrhea, dyspnea, depression, and lameness. The RDS could range from 0 to 6, with the following criteria: score 0 means normal (no coughing), score 1 means mild coughing after stimulated exercise, score 2 means mild coughing at rest, score 3 means moderate coughing after stimulated exercise, score 4 means moderate coughing at rest, score 5 means severe coughing after stimulated exercise, and score 6 means severe coughing at rest.

### Macroscopic and microscopic lung lesions

At necropsy (D35/D49), the lungs were removed and evaluated for macroscopic *Mycoplasma*-like lesions using a scoring method as described [24]. The lesion score for each lung ranged from 0 (no *Mycoplasma*-like lesions) to 35 (entire lung affected).

Additionally, tissue samples were collected from the top of the left apical, cardiac, and diaphragmatic lobes for histopathological analysis. These samples were fixed in 10% neutral-buffered formalin and subsequently embedded in paraffin before being stained with hematoxylin and eosin. Microscopic examination was performed using a Nanozoomer NDP slide scanner (Hamamatsu Photonics, Hamamatsu, Japan) with the associated NDP.View2 platform. The degree of peribronchiolar and perivascular lymphohistiocytic infiltration and nodule formation (cuffing) was assessed in ten random microscopic fields (at 40× magnification) from each sample, following a previously described scoring system [25]. The lesion severity score ranged from 1 to 5, with the following classifications: score 1 means limited infiltration of macrophages and lymphocytes around bronchioles, with no cellular exudates in the airways and alveolar spaces; score 2 means mild to moderate infiltrates with diffuse cellular exudates into the airways; score 3–5 means lesions characteristic of broncho-interstitial pneumonia, with severity increasing from mild to severe. These lesions are centered around the bronchioles and extend into the interstitium, characterized by lymph follicular infiltration and mixed inflammatory cell exudates. Scores 3, 4, and 5 were considered indicative of infection with *M. hyopneumoniae*. All these analyses were performed in a blinded manner.

### Quantitative PCR for *M. hyopneumoniae* DNA in BALF

A commercial kit (DNeasy^®^ Blood & Tissue kit, Qiagen, Venlo, The Netherlands) was used to extract DNA from the BALF samples. Then, a quantitative PCR (qPCR) was performed to measure the *M. hyopneumoniae* DNA load as described previously [26]. Briefly, 1 µL DNA sample was mixed with iQ Supermix (5 µL), nuclease-free water (2.7 µL), forward primer (20 µM, 0.5 µL), reverse primer (20 µM, 0.5 µL), and a probe (10 µM, 0.3 µL) in a total volume of 10 µL for DNA analysis. The threshold values were converted to the number of organisms using a tenfold dilution series of *M. hyopneumoniae* F7.2C DNA. Samples were considered negative if the values were below the highest dilution (1.50 × 10^1^/mL; 1.18 log copies/mL). The *M. hyopneumoniae* load was expressed as log_10_ genome copies/µL.

### *M. hyopneumoniae*-specific antibody responses in serum and BALF

The number of *M. hyopneumoniae* seropositive animals was determined using a commercial blocking enzyme-linked immunosorbent assay (ELISA; IDEIA™ *Mycoplasma hyopneumoniae* EIA kit, Oxoid Limited, Hampshire, UK), following the manufacturer’s instructions. Samples with optical density (OD) lower than 50% of the average OD of the buffer control were considered positive, while those with OD higher than 50% of the average OD of the buffer control were considered negative.

*M. hyopneumoniae*-specific immunoglobulin G (IgG) and immunoglobulin A (IgA) were measured in both serum (diluted 1:200 for IgG and 1:10 for IgA) and BALF (diluted 1:10 for both) using an in-house indirect ELISA with Tween 20-extracted *M. hyopneumoniae* antigens [27]. All samples were tested in duplicate. If the OD difference between the duplicates exceeded 0.05 or represented more than 25% of the OD value, the samples were retested. Samples were considered positive for both BALF and serum if their average OD value was greater than the cutoff, which was calculated as the average OD of the NC group plus three times the standard deviation (SD) of the group [4].

### Cytokines in BALF

The concentrations of tumor necrosis factor alpha (TNF-α), interleukin (IL)−1β, IL-6, IL-10, and the chemokine CXCL-8 in BALF were quantified using a multiplex immunoassay (Custom Porcine Procarta Plex Multiplex Immunoassay, ThermoFisher Scientific, Waltham, MA, USA), according to the manufacturer’s instructions. BALF samples were diluted at a ratio of 1:2 prior to analysis. Data analysis was performed as described previously [17].

The concentration of IL-17A in BALF was measured using a commercially available ELISA kit (ThermoFisher Scientific, Waltham, MA, USA) according to the manufacturer’s protocol. BALF samples were diluted at a ratio of 1:2 prior to analysis. The OD was measured at 450 nm using a microtiter plate reader (Multiskan GO, ThermoFisher Scientific).

### T cell assays

To assess cytokine production by T cells, peripheral blood mononuclear cells (PBMCs) were isolated and collected in blood samples taken at different time points, namely D0, D7, D21, D35, and D49. Lymphocytes were also collected from bronchial lymph nodes at euthanasia on D35 and D49. The cells were subsequently stimulated and stained for surface markers and intracellular cytokines as described [4, 18]. Briefly, 2.5 × 10^6^ cells were seeded in 24-well plates and stimulated for 20 h with 3.125 × 10^7^ CCU of inactivated *M. hyopneumoniae* strain F7.2C in 0.5 mL of AIM-V medium (Gibco™, ThermoFisher Scientific, Waltham, MA, USA). Stimulation with concanavalin A (10 µg/mL; Sigma-Aldrich) served as a positive control, while AIM-V medium alone was used as a negative control. To investigate cytokine production, protein secretion was inhibited by adding 1 µL Brefeldin A (eBioscience, San Diego, CA, USA) for the last 4 h of stimulation. Following stimulation, cells were collected, incubated with a LIVE/DEAD™ Fixable Aqua Dead Cell Stain Kit (ThermoFisher Scientific) and surface stained using anti-CD3 DyLight755 (clone PPT3, in house), anti-CD4 (clone 74-12-4, in house), and anti-CD8α (clone 11–295-33, in house) mAbs, along with their corresponding secondary antibodies: anti-mouse IgG2b fluorescein isothiocyanate (FITC) (BioLegend, San Diego, CA, USA) and anti-mouse IgG2a PE-Cy7 (Abcam, Cambridge, UK). For intracellular cytokine detection, cells were fixed and permeabilized (BD Fix/Perm, Becton Dickinson, Franklin Lakes, NJ, USA) and then stained with anti-human TNF-α AlexaFluor 647 (clone Mab11; BioLegend), anti-porcine IFN-γ PerCP-Cy5.5 (clone P2G10; BD Pharmingen™, Becton Dickinson, Franklin Lakes, NJ, USA), and anti-human IL-17A PE (clone SCPL1362; BD Pharmingen™). Samples were acquired using a CytoFLEX flow cytometer (Beckman Coulter, Brea, CA, USA), and data were analyzed using CytExpert software (Beckman Coulter). The gating strategy was identical to that described previously and is shown in Additional file 4 [17].

In addition to cytokine profiling, cell proliferation was evaluated using a previously established protocol with minor modifications [4]. PBMCs and bronchial lymph node cells were labeled using a Cell Trace™ Cell Proliferation Kit (ThermoFisher Scientific) following the manufacturer’s instructions. Labeled cells (1 × 10^6^/well) were seeded in 24-well plates in 0.5 mL cell culture medium (Dulbecco’s modified Eagle medium (DMEM), 10% fetal calf serum (FCS), 1% penicillin/streptomycin, 1% non-essential amino acids) and stimulated for 87 h with 3.125 × 10^7^ CCU of the inactivated *M. hyopneumoniae* F7.2C strain. Concanavalin A (10 µg/mL) served as a positive control, while culture medium was used as a negative control to account for background proliferation. Upon stimulation, cells were collected and stained to detect CD3, CD4, and CD8α surface expression as described [4]. Propidium iodide staining was performed to exclude dead cells. The gating hierarchy was as described previously and is shown in Additional file 4 [4]. A minimum of 1000000 events were recorded for the intracellular cytokine staining (ICS) assay, and 500000 events were recorded for the proliferation assay.

### Immunohistochemistry of lung tissue

Immunohistochemical staining (IHC) for IgA was performed in the lung tissue of pigs necropsied on D35 and D49. Paraffin-embedded lung samples were cut into 5-µm sections and mounted on 3-aminopropyltriethoxysilane-coated slides. The sections were dewaxed and rehydrated, followed by 20 min of heat-induced epitope retrieval in EDTA buffer (pH 9.0). Next, they were incubated with polyclonal rabbit anti-human IgA heavy chain antibody (ProteinTech, Ref. 11449-1-AP) in primary antibody dilution buffer containing background reduction components (Agilent, Ref. S302283-2) at a dilution of 1:5000 for 15 min. Signal detection was performed using the Bond Polymer Refine Detection Kit (Leica, Ref. DS9800) according to the manufacturer’s instructions, with 3,3′-diaminobenzidine (DAB) staining and hematoxylin counterstaining. The sections were dehydrated, mounted, and examined under a microscope using a 40 × magnification. Images were captured using Leica imaging software. Five randomly selected fields were examined from each section, and the IgA-positive area was calculated using ImageJ software, expressing it as the percentage of IgA-positive area relative to the total tissue area.

### Statistical analyses

Graphical presentations of the data were prepared using GraphPad Prism 10.0 (GraphPad Software, San Diego, CA, USA), and the data were analyzed using IBM SPSS Statistics software version 29 (IBM Corp., Armonk, NY, USA). A Mann–Whitney *U* test was used to compare the Respiratory Disease Score (RDS), macroscopic lung lesion score (MLCL), microscopic lung lesion score (MLL), the percentage of various cell subsets, the cytokine responses, and IgA in lung tissue between the two groups. The log_10_-transformed DNA loads of *M. hyopneumoniae* between the two groups were compared using LMM and a Mann–Whitney *U* test. The *M. hyopneumoniae*-specific antibody responses in serum and BALF (IgA and IgG) were analyzed using different tests. Binary crosstab analyses were used to examine frequency distributions and correlations of IgG-related parameters. Group differences in *M. hyopneumoniae*-specific antibody in serum were assessed using chi-squared tests. For IgA-related parameters in serum and BALF, generalized linear models (GLMs) were employed to determine the effects of independent variables on dependent outcomes. The correlations between the DNA load of *M. hyopneumoniae* in BALF, antibodies in serum and BALF, cytokines in BALF or %IgA + area in lungs on D49, and infection severity (RDS: from D22-49; MLCL and MLL: D49) in the C or V group were assessed using Pearson’s correlation analysis with a two-tailed test (GraphPad Prism software 10.0). The Pearson correlation coefficient (*r*) and its 95% confidence interval (CI) were calculated, and the coefficient of determination (*R*^2^) is reported to evaluate the strength of the association. The strength of the correlation can be classified on the basis of the coefficient of determination (${R}^{2}$) as follows: values below 0.3 indicate a weak correlation; values between 0.3 and 0.7 indicate a moderate correlation; values between 0.7 and 0.9 indicate a strong correlation; and values above 0.9 indicate a very strong correlation. The NC group was not included in the statistical analyses, as this group only served as a sentinel group to prove that the pigs were free of *M. hyopneumoniae*. Statistical results were considered significant when *P* ≤ 0.05.

## Results

### Clinical signs, lung lesions, and *M. hyopneumoniae* load

Clinical signs combined with laboratory analysis constitute the current standard diagnostic approach for porcine *M. hyopneumoniae* [28]. Most studies demonstrated that vaccines alleviate clinical signs and to a certain degree also eliminate *M. hyopneumoniae* from the host [17, 27]. In this study, vaccination significantly reduced the RDS from D36 to D49 (*P* = 0.03) as well as the *M. hyopneumoniae* DNA load in BALF on D49 (*P* = 0.001) as compared with the C group (Table 1). At the macroscopic level, darker-red and solid pulmonary consolidation areas at the apex of the lung was found in the C group. At the microscopic level, a large number of infiltrated neutrophils and proliferated epithelial cells were found. However, the differences for these two parameters were not statistically different between the groups (*P* = 0.142) (Table 1). Moreover, a moderate positive correlation was observed between the DNA load of *M. hyopneumoniae* and RDS (D22-D49) (*R*^2^ = 0.3644, *P* = 0.0223) or MLCL (*R*^2^ = 0.4094, *P* = 0.0137) in the V group (Additional file 3). The sentinel animals (NC group) remained negative for *M. hyopneumoniae* DNA throughout the study. Thus, vaccination reduced coughing and the *M. hyopneumoniae* DNA load in BALF upon infection.

**Table 1:** **Clinical signs and** ***M. hyopneumoniae*** **DNA load at different timepoints in control and vaccinated group.**

Clinical parameters					
Parameters/group	Period or timepoint	C (*n*)		V (*n*)	
		Average	Median	Average	Median
RDS	D1 to D21	0.01 (20)	0.00 (20)	0.05 (20)	0.00 (20)
	D22 to D35	1.20 (20)	1.11 (20)	1.39 (20)	1.04 (20)
	D36 to D49	2.49 (16)^a^	2.07 (16)	1.38 (14)^b^	1.04 (14)
	D22 to D49	1.90 (16)	1.55 (16)	1.43 (14)	1.00 (14)
MLCL	D49	7.64 (16)	6.51 (16)	4.12 (14)	2.81 (14)
MLL	D49	2.28 (16)	2.20 (16)	2.02 (14)	2.03 (14)
Log_10_ *M. hyo* DNA/µL (qPCR)	D35	4.45 (19)	4.13 (19)	4.63 (18)	4.29 (18)
	D49	5.11 (16)^a^	4.98 (16)	4.96 (14)^b^	4.60 (14)

C: not vaccinated but infected; V: vaccinated and infected. Pigs were vaccinated once with an inactivated *M. hyo* vaccine. (*n*) = number of animals in each group

D0: vaccination; D21: experimental infection; D35: necropsy of 4 animals in each group; D49: necropsy of the remaining animals

RDS: Respiratory Disease Score (RDS) was assessed daily

MLCL: Macroscopic lung lesion score was determined on D35 and D49

MLL: Microscopic lung lesions were assessed on D35 and D49

^a,b^ Within a column, values with different superscript letters are significantly (*P* < 0.05) different.

### Vaccination enhanced *M. hyopneumoniae*-specific antibody responses in serum and BALF

**Figure 2:**
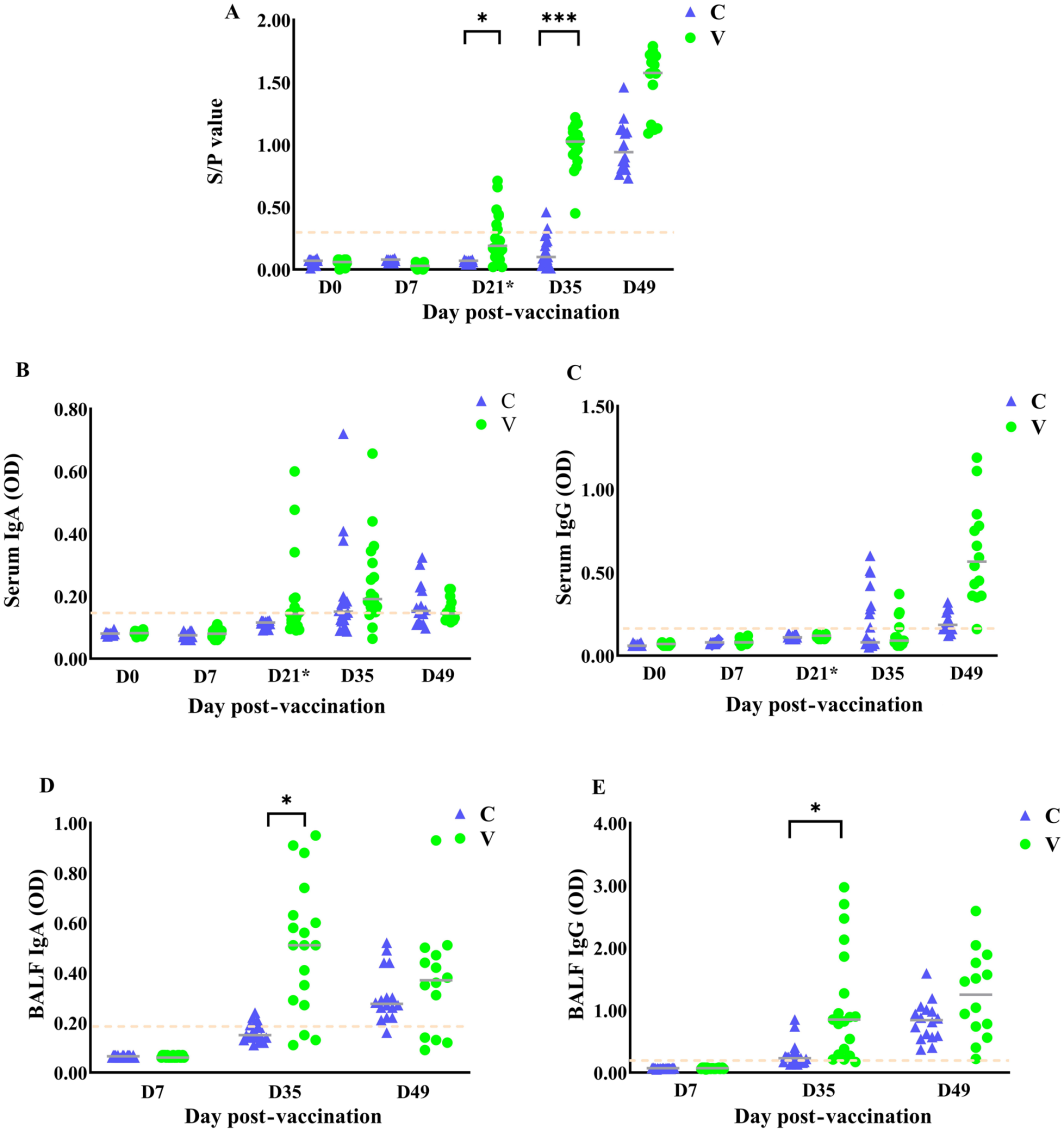
**Vaccine-induced antibody responses in serum and BALF of pigs vaccinated against *M. hyopneumoniae* on day 0 (D0), and experimentally infected on D21.** C: non-vaccinated and challenged; V: vaccinated and challenged. D35: 2 weeks after challenge (necropsy of four animals in each group); D49: 4 weeks after challenge (necropsy of the remaining animals). Blood was collected on D0, D7, D21, D35, and D49. BALF was collected on D7, D35, and D49. **A** *M. hyopneumoniea*-specific antibodies in serum in the different treatment groups. The dashed pink line indicates the cutoff value (S/P ratio ≤ 0.30). **B**, **C** Serum IgA and IgG levels following vaccination and challenge infection. **D**, **E** BALF IgA and IgG levels following vaccination and challenge infection. The dashed light-orange lines indicate the cutoff value, which was calculated as the average OD of the samples of the NC group + three times the standard deviation. The solid gray line represents the median. Values are expressed as OD. An asterisk (*) over the bar indicates a significant difference (* indicates *p* ≤ 0.05, ** indicates *p* ≤ 0.01, *** indicates *p* ≤ 0.001, and **** indicates *p* ≤ 0.0001).

Previous studies showed that most *M. hyopneumoniae* vaccines induced humoral immune responses in pigs [17, 21, 29]. In this study, the antibody levels in serum and BALF were measured to determine whether vaccination triggered antibody responses (Figure 2). All pigs from the NC group tested negative during the whole period. After vaccination, all pigs (*n* = 20) from the C group tested negative. In the V group, all pigs (*n* = 20) tested negative on D0 and D7, while seven pigs seroconverted on D21, which differed significantly from the C group (*P* = 0.04) (Figure 2A). After challenge infection, all animals in the V group seroconverted in contrast to only two animals in the C group on D35 (*P* < 0.001). Antibody levels further increased till D49. As anticipated, serum antibody levels were induced following vaccination and increased following infection.

An in-house ELISA was carried out on serum and BALF samples to further investigate the vaccine-induced IgG and IgA levels (Figure 2B–E). *M. hyopneumoniae*-specific IgA and IgG levels in serum and BALF of the NC group were below the cutoff value at all timepoints. *M. hyopneumoniae*-specific serum IgA and IgG levels from the C and the V group were below the cutoff value on D0 and D7 (Figure 2B, C). In serum at D21, there was no significant difference between groups. However, in BALF at D35, the V group had significantly higher IgA (*P* = 0.005) and IgG (*P* = 0.02) levels as compared with the control group (C) (Figure 2D, E).

### Vaccination reduced local cytokine production

**Figure 3:**
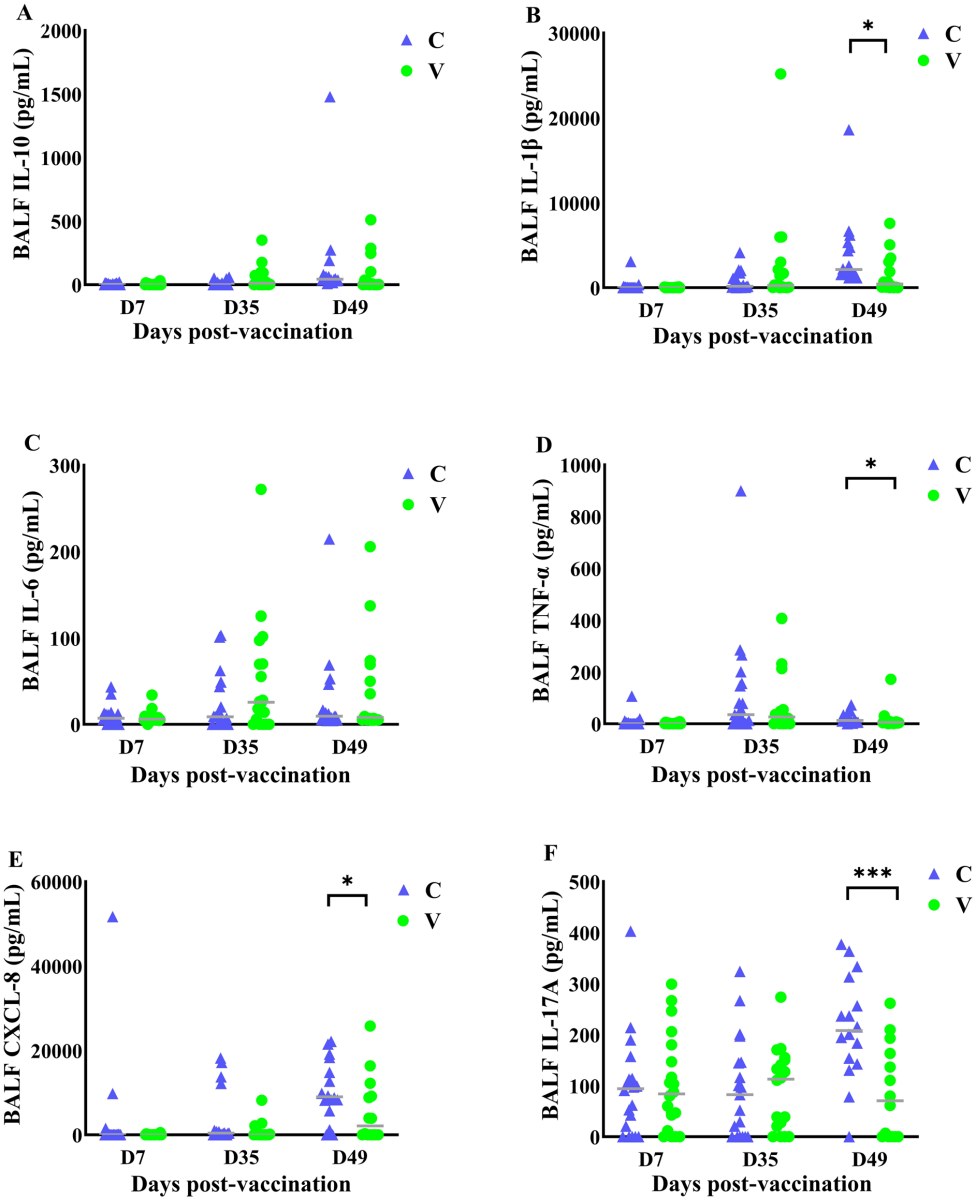
**Cytokines in the bronchoalveolar lavage fluid (BALF) of pigs vaccinated against *M. hyopneumoniae* on day 0 (D0), and experimentally infected on D21.** C: non-vaccinated and challenged; V: vaccinated and challenged. D35: two weeks after challenge (necropsy of four animals in each group); D49: 4 weeks after challenge (necropsy of the remaining animals). BALF was collected on D7, D35, and D49. The concentrations (pg/mL) of IL-10 (**A**), IL-1β (**B**), IL-6 (**C**), TNF-α (**D**), and CXCL-8 (**E**) were determined using a multiplex immunoassay. The concentration of IL-17A (F) was determined with a commercial ELISA. The solid gray line represents the median. D: days after vaccination. An asterisk (*) over the bar indicates a significant difference (* indicates *p* ≤ 0.05, ** indicates *p* ≤ 0.01, *** indicates *p* ≤ 0.001, and **** indicates *p* ≤ 0.0001).

In addition to alterations in antibody levels, vaccination against *M. hyopneumoniae* also induced changes in local cytokine production [27]. The concentration of different cytokines in BALF was measured in the different groups at different timepoints to understand whether the combination vaccine triggered a similar response as reported previously for *M. hyopneumoniae* vaccines (Figure 3A–F). The concentrations of all cytokines in the NC group were lower than in the C and V groups during the whole period. No differences in concentration of cytokines in BALF were observed between the C and V groups on D7 and D35. On D49, the concentration of IL-1β was also significantly higher in the C group as compared with the V group (*P* = 0.015), while IL-6 levels did not differ between both groups. Furthermore, the concentrations of TNF-α, CXCL-8, and IL-17A on D49 were also significantly higher in the C group compared with the V group (TNF-α, *P* = 0.043; CXCL-8, *P* = 0.027; IL-17A, *P* < 0.001). The concentration of IL-10 differed between these two groups, albeit not significantly (*P* = 0.07). These results indicate that vaccination reduced the levels of IL-1β, TNF-α, CXCL-8, and IL-17A in BALF at 4 weeks after challenge infection.

To understand whether these cytokine levels were associated with disease severity, we performed correlation analyses. A weak positive correlation was observed between the IL-1β concentration (D49) and RDS (D22-D49) in the C group (*R*^2^ = 0.2881, *P* = 0.0321) and a moderate positive correlation between the IL-1β concentration and RDS (D22-D49) (*R*^2^ = 0.5079, *P* = 0.0042) and MLCL (D49) (*R*^2^ = 0.6655, *P* < 0.0001) in the V group (Additional file 3). Moreover, a weak positive correlation was also found between the IL-17A concentration (D49) and MLL (D49) in the C group (*R*^2^ = 0.2122, *P* = 0.0544) (Additional file 3).

### Vaccination triggered cell-mediated immunity

**Figure 4:**
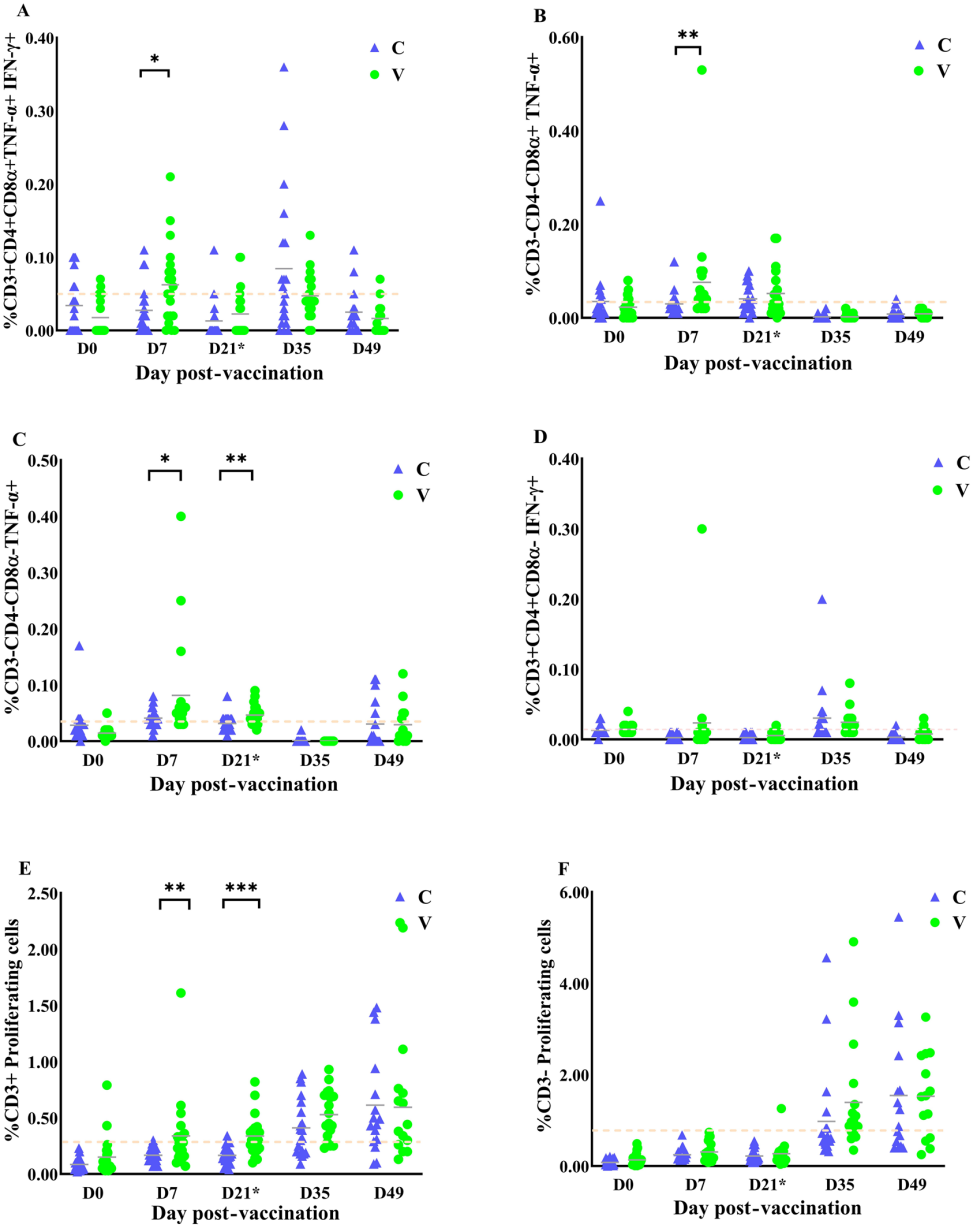
**The percentages of *M. hyopneumoniae*-specific cytokine-producing immune cells and proliferation in blood of pigs vaccinated against *M. hyopneumoniae* on day 0 (D0), and experimentally infected on D21.** C: non-vaccinated and challenged; V: vaccinated and challenged. D35: 2 weeks after challenge (necropsy of 4 animals in each group); D49: 4 weeks after challenge (necropsy of the remaining animals). Blood was collected on D0, D7, D21, D35, and D49. D21* means pigs were experimentally infected on D21. **A**–**F** The percentage of *M. hyopneumoniae*-specific cytokine-differentiating CD3^+^CD4^+^CD8α^+^ TNF-α^+^/IFN-γ^+^ cells, CD3^−^CD4^−^CD8α + TNF-α^+^ cells, CD3^−^CD4^−^CD8α^−^ TNF-α^+^ cells, CD3^+^CD4^+^CD8α^−^ IFN-γ^+^ cells, and proliferating CD3^+^ cells and CD3^−^ cells in blood. The solid gray line represents the median; samples were considered positive if the percentage values exceeded the cutoff value (light-orange dotted lines). The cutoff value was calculated as the average value of the negative control group (NC) at each time point. Values are expressed as percentages (%). An asterisk (*) over the bar indicates a significant difference (* indicates *p* ≤ 0.05, ** indicates *p* ≤ 0.01, *** indicates *p* ≤ 0.001, and **** indicates *p* ≤ 0.0001).

Elucidating the activation of immune cell populations in peripheral tissues and lymph nodes is critical for evaluating the immunogenicity of *M. hyopneumoniae* vaccines [4, 17, 30]. On this basis, we evaluated cytokine production by various T cell subsets in recall assays (Figure 4). Consistent with expectations, T cells from all pigs exhibited cytokine production following concanavalin A stimulation (data not shown). After vaccination, the percentage of IFN-γ^+^/TNF-α^+^ CD4^+^CD8α^+^ T cells (*P* = 0.026) (Figure 4A) and TNF-α^+^ CD3^−^CD4^−^CD8α^+^ cells (*P* = 0.002) (Figure 4B) in PBMCs were significantly higher in the V group than in the C group on D7. The percentage of TNF-α^+^ CD3^−^CD4^−^CD8α^−^ cells was also significantly higher in the V group on D7 (*P* = 0.028) and D21 (*P* = 0.005) (Figure 4C). Furthermore, the proportion of proliferating CD3^+^ T cells in PBMCs was elevated in the V group compared with the C group on D7 (*P* = 0.008) and D21 (*P* < 0.001) (Figure 4E). Overall, vaccination induced the activation of IFN-γ^+^/TNF-α^+^ CD4^+^CD8α^+^ T cells, TNF-α^+^ CD3^−^CD8α^+^ cells and TNF-α^+^ CD3^−^CD4^−^CD8α^−^ as well as the proliferating CD3^+^ T cells in blood after vaccination, while it may also reduce IFN-γ^+^ CD4^+^ T cells in blood 2 weeks after challenge (Figure 4).

**Figure 5:**
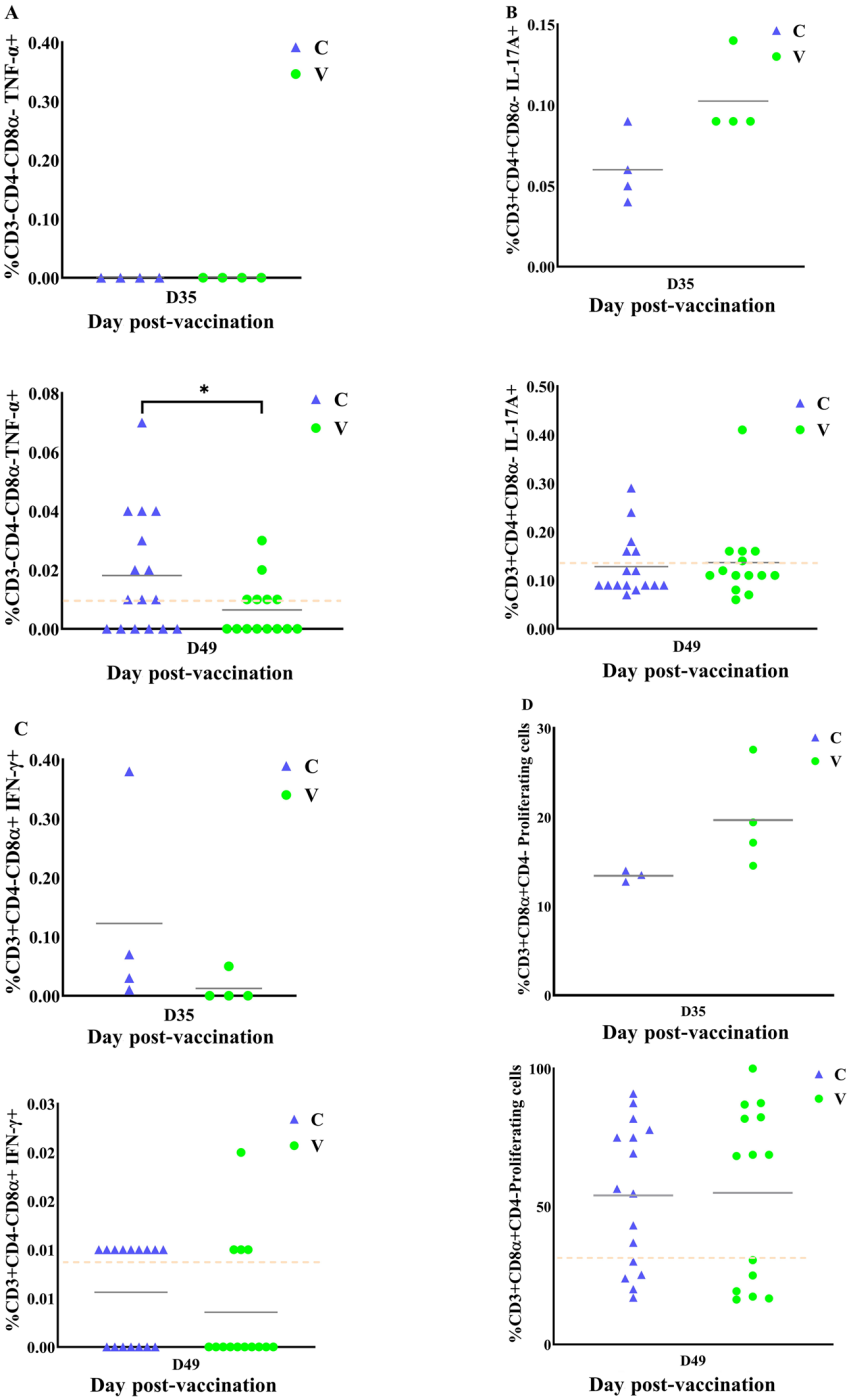
**The percentages of *M. hyopneumoniae*-specific cytokine-producing immune cells and proliferation in bronchial lymph nodes (BLNs) of pigs vaccinated against *M. hyopneumoniae* on day 0 (D0), and experimentally infected on D21.** C: non-vaccinated and challenged; V: vaccinated and challenged. D35: 2 weeks after challenge (necropsy of 4 animals in each group); D49: 4 weeks after challenge (necropsy of the remaining animals). BLNs were collected on D35 and D49. D21* means pigs were experimentally infected on D21. **A**–**D** show the percentage of *M. hyopneumoniae-*specific cytokine-differentiating CD3^−^CD4^−^CD8α^−^ TNF-α^+^ cells, CD3^+^CD4^+^CD8α^−^ IL-17A^+^ cells, CD3^+^CD4^−^CD8α^+^ IFN-γ^+^ cells, and proliferating CD3^+^CD8α^+^ cells in BLNs. The solid gray line represents the median; samples were considered positive if the percentage values exceeded the cutoff value (light-orange dotted lines). The cutoff value was calculated as the average value of the negative control group (NC) on D49. Values are expressed as percentages (%). An asterisk (*) over the bar indicates a significant difference (* indicates *p* ≤ 0.05, ** indicates *p* ≤ 0.01, *** indicates *p* ≤ 0.001, and **** indicates *p* ≤ 0.0001).

When evaluating the presence of *M. hyopneumoniae*-specific T cells in the local draining lymph nodes, no significant differences were observed between the groups on D35. On D49, the percentage of TNF-α^+^ CD3^−^CD4^−^CD8α^−^ cells was significantly higher in the C group as compared with the V group (*P* = 0.016) (Figure 5A). While the V group showed a trend toward a higher presence of IL-17A^+^ CD3^+^CD4^+^CD8α^−^ cells compared with the C group (Figure 5B), the C group tended to exhibit a higher presence of IFN-γ^+^ CD3^+^CD4^−^CD8α^+^ cells (Figure 5C). In addition, the V group showed an increasing trend in the percentage of proliferating CD3^+^CD8α^+^ T cells as compared with the C group on D35 (Figure 5D). This indicated that, upon vaccination and challenge infection, *M. hyopneumoniae*-specific T cells are present in the bronchial lymph nodes 2 weeks after the challenge infection.

### Local mucosal immunity was induced in both groups upon infection

**Figure 6:**
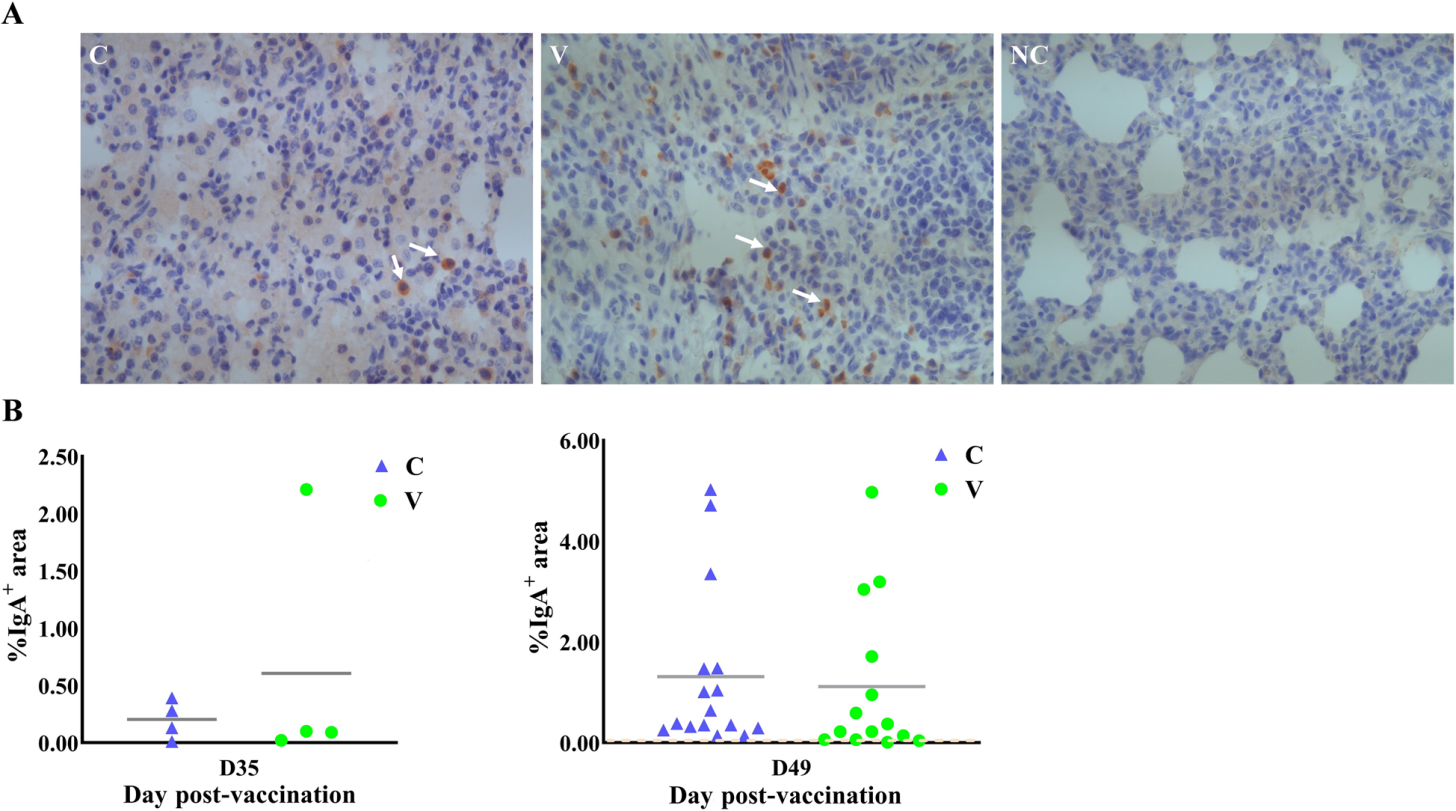
**Immunohistochemical detection of IgA in lung tissue of pigs on D35 and D49. **Pigs of the V group were vaccinated against *M. hyopneumoniae* on day 0 (D0). Pigs of the V and C groups were experimentally infected on D21. C: non-vaccinated and challenged; V: vaccinated and challenged. **A** Representative staining from each group; piglets infected with *M. hyopneumoniae*, displaying numerous IgA-positive area (brown staining). White arrows indicate positive signals. **B** Percentage of positive IgA^+^ area in each group of lung tissues. The solid gray line represents the average; samples were considered positive if their percentage values exceeded the cutoff value. The light-orange dotted line represents the cutoff value, which was calculated as the average value of the negative control group (NC, 0.04%). Values are expressed as % of IgA^+^ area.

Some studies on porcine respiratory diseases have demonstrated that IgA responses in vaccinated animals only appear following challenge infection [31, 32]. Therefore, the percentage of IgA antibody positivity in lung tissue from the C and V groups was measured on D35 and D49 in our study. Although the percentage of the IgA^+^ area in both groups was higher than that observed in the NC group, no significant difference was detected between the two groups on D49 (Figure 6). There was however a weak positive correlation between the %IgA + area and MLCL (*R*^2^ = 0.2746, *P* = 0.0372) (Additional file 3).

## Discussion

This study investigated the immune responses upon vaccination of 24-day-old weaned piglets with a novel combined vaccine consisting of an inactivated *M. hyopneumoniae* vaccine with the capsid protein of PCV2d. Previously, we tested the immunogenicity of Hyogen, commercial *M. hyopneumoniae* vaccine, showing that it alleviated clinical signs, enhanced serum antibodies, and induced cytokines in BALF and peripheral blood [17]. In addition, another commercial *M. hyopneumoniae* vaccine induced long-lasting cell-mediated immunity even in the absence of detectable antibodies, highlighting the importance of cellular immune responses [21]. Furthermore, an inactivated vaccine candidate using an Mhp chimeric protein (P97R1-P46-P42) fused with CD40L as a molecular adjuvant enhanced antibody responses, lymphocyte proliferation, and IL-4 levels compared with conventional vaccines [33]. These studies collectively established a foundation for understanding *M. hyopneumoniae* vaccine-induced immunity. However, none investigated tissue-level immune responses as in the present study. Combined vaccines can be more easily implemented in farm health management practices and are also better for animal welfare and biosecurity as fewer injections are needed. Previous studies have highlighted the advantages of combined vaccines targeting *Mycoplasma hyopneumoniae* and PCV2d in enhancing antibody responses and demonstrating safety [8–10, 22]. Concerning the mechanism involved in the increased *M. hyopneumoniae* antibody titers in BALF upon immunization and challenge infection, the prime–pull mechanism may play an important role. This prime–pull approach is often used to induce mucosal immune responses. In this approach, the systemic immune system is primed with a parenteral immunization, which triggers vaccine-specific lymphocytes at the local draining lymph nodes [34]. These vaccine-specific lymphocytes then circulate through the vascular system and populate the other lymph nodes. The push then consists of a mucosal immunization or, in our case, a challenge infection that recruits the lymphocytes to the site of the booster immunization or the challenge infection [35–37]. The present study focused on evaluating the immunogenicity and efficacy of this vaccine against *M. hyopneumoniae* challenge infection.

Three weeks post-vaccination, the piglets were experimentally infected with *Mycoplasma hyopneumoniae* strains F7.2C and F1.12A [18, 27, 35–37]. Clinical signs and immune responses were monitored throughout the study, and vaccinated and non-vaccinated animals were necropsied at weeks 2 and 4 post-infection to assess early and late responses. The data showed that, 1 week post-vaccination, the V group exhibited significantly increased levels of IFN-γ^+^/TNF-α^+^ CD4^+^CD8α^+^ T cells, TNF-α^+^ CD3^−^CD4^−^CD8α^+^ cells, and proliferative T cells in blood, with this effect persisting through 3 weeks post-vaccination. Furthermore, at 2 weeks after challenge, a markedly higher proportion of proliferative T cells was detected in peripheral blood and the bronchial lymph nodes of vaccinated animals. At 4 weeks post-infection, vaccinated animals exhibited a significantly lower proportion of TNF-α^+^ CD3^−^CD4^−^CD8α^−^ cells. This indicates that the combined vaccine induced both local and systemic cell-mediated immunity. Concurrently, vaccinated animals demonstrated higher serum IgA and IgG levels, lower *M. hyopneumoniae* DNA load in BALF, and milder clinical signs.

Previous studies have shown that vaccination may not only reduce clinical signs but also reduce the number of pathogens in the respiratory tract, thereby improving performance [1, 18, 38]. Our study showed that the RDS from D35 to D49 was lower in vaccinated animals, and that the *M. hyopneumoniae* load was also reduced on D49. Although macroscopic lung lesion and microscopic lung lesion on D49 did not significantly differ between the two groups, vaccination still resulted in lower scores, which is consistent with findings reported in previous research [39]. *M. hyopneumoniae* infections cause chronic disease, and the lung lesions usually peak 2–4 weeks after infection [1]. The reduced clinical signs and *M. hyopneumoniae* DNA load in the vaccinated animals indicated that the vaccine is efficacious and may also reduce the possibility of transmission of *M. hyopneumoniae* [11, 40, 41]. Moreover, the *M. hyopneumoniae* DNA load correlated with the RDS (D22-D49) in the V group, suggesting that lower bacterial burden may contribute to less severe respiratory disease progression.

Intramuscular injection of commercial inactivated vaccines can induce the production of specific antibodies in the serum and the respiratory tract [39, 42]. This study showed that a single administration of the vaccine increased the serum levels of *M. hyopneumoniae*-specific antibodies on D21. In addition, IgA and IgG antibodies in the BALF of the vaccine group were higher than those of the control group 2 weeks after infection, which is also similar to a previous study [39]. This confirms that the vaccine might prime the mucosal immune system, resulting in an increased presence of antibodies in the BALF upon subsequent infection, and thus may play a role in reducing the number of *M. hyopneumoniae* copies [12, 39, 43]. A previous study has shown that local mucosal antibodies may play a role in preventing *M. hyopneumoniae* from adhering to the ciliated epithelium of the respiratory tract [39]. Another study, however, could not demonstrate an association between the IgA levels in the lung and protection [20]. Here, the percentage of IgA^+^ area in lung tissue weakly correlated with MLCL, implying that local mucosal humoral responses may increase in parallel with lesion severity. This may reflect a compensatory immune response aiming to control pathogen colonization at the respiratory mucosal surface. Future studies should investigate earlier time points after infection in more animals to investigate the impact of vaccination and infection on IgA levels in lung tissues.

Previous studies have indicated that inactivated vaccines can provide partial protection against *M. hyopneumoniae* infection and colonization of the lower respiratory tract by modulating the inflammatory responses [30, 44, 45]. This study showed that the concentrations of IL-1β, CXCL-8, TNF-α, and IL-17A in the BALF of vaccinated animals were lower than in non-vaccinated animals on D49, and IL-10 was numerically lower in the non-vaccinated animals on D49. For the pro-inflammatory cytokines, these are consistent with previous results from our group [17, 27]. IL-10 is a typical anti-inflammatory factor that can act as a negative feedback mechanism to limit tissue damage [16]. Our results on IL-10 production in BALF differ from those in a previous study [17], which may be related to differences in infection conditions or individual variation. Further investigation is needed to clarify this discrepancy. CXCL-8 plays a key role in neutrophil recruitment during *M. hyopneumoniae* infection. In our study, CXCL-8 levels were lower in the vaccinated animals than in the non-vaccinated animals, suggesting that vaccination mitigated the excessive inflammatory response in the lung while maintaining effective immune control of the pathogen [46]. The increase of these cytokines in BALF points to an inflammatory response and related tissue damage [16, 47–49]. In addition, IL-1β levels are associated with disease severity. In the C group, a weak positive correlation between IL-1β concentration and RDS was observed, whereas in the V group, moderate correlations were observed between IL-1β levels and both RDS and MLCL. These results indicate that IL-1β may be closely linked to inflammatory processes involved in lesion development, and its elevated levels could reflect enhanced host inflammatory responses associated with increased disease severity. Additionally, a weak positive correlation was identified between IL-17A concentration and MLL in the C group, suggesting that IL-17A may also participate in the inflammatory response during infection.

Vaccine-induced cellular immune responses are also reflected in the cytokine production and proliferation of different T cell subsets, mainly including CD4^+^ T cells, CD8α^+^ T cells and CD4^+^CD8α^+^ T cells. These T cell subsets are considered important for protection against *M. hyopneumoniae* infection [27]. Commercial inactivated vaccines have also been shown to induce different T cell subsets in blood [17, 20, 50–52]. The present results on different T cell subsets in the blood showed that, just 1 week after immunization, IFN-γ^+^/TNF-α^+^ CD3^+^CD4^+^CD8α^+^ cells were detected, while proliferating T cells were detected at both 1 and 3 weeks after vaccination. These findings suggest that the vaccine elicited polyfunctional, antigen-experienced CD4^+^CD8α^+^ T cell responses, which are indicative of an effective cellular immune activation [14, 25]. In addition, TNF-α^+^ CD3^−^CD4^−^CD8α^+^ cells (natural killer (NK) cells) were induced in vaccinated animals on D7 and D21, respectively. NK cells are characterized by their ability to kill cells infected with intracellular pathogens and their production of IFN-γ and TNF-α upon their activation [53]. Whether these NK cells can recognize antigens or pathogen recognition receptor ligands associated with *M. hyopneumoniae* or if they are induced by cytokines in the recall assay remains unresolved. Furthermore, TNFα^+^ CD3^−^CD4^−^CD8α^−^ cells were also induced upon vaccination. These cells most likely represent innate B cells, which create a suitable microenvironment for T cell responses and memory formation [17, 47–50, 54]. These results suggest that the vaccine induced a cell-mediated immune response.

Studies on H1N1 influenza A virus infection have shown that the immune response in BLNs may be closely related to the clearance of the pathogen and resolution of lesions [55]. The present study showed that, 4 weeks after challenge, the TNF-α^+^ CD3^−^CD4^−^CD8α^−^ cells (B cells) in BLNs of vaccinated animals were lower than in the non-vaccinated animals. In murine infection models, B cells have been identified as an important source of TNF-α, which contributes to CD4^+^ T cell expansion and pathogen clearance [56, 57]. Although there was no difference between the groups at D35, we still found that the IL-17A-producing CD4^+^CD8α^−^ T cells and proliferating CD8α^+^ CTLs in the BLNs were higher in vaccinated than in non-vaccinated animals. IL-17A is the hallmark cytokine produced by Th17 cells that promotes immune cell mobilization from bone marrow and neutrophil recruitment to sites of infection. In the lung, IL-17A activates airway epithelial cells to produce chemokines such as CXCL-8 to attract neutrophils and to induce local inflammation [58–60]. In contrast, IL-17A^+^ CD4^+^CD8α^−^ T cells were detected in BLNs at 2 weeks after challenge in this study, which suggests that antigens had been transported to the BLNs through antigen-presenting cells, such as dendritic cells, inducing local IL-17A secretion. The proliferation of CD8α^+^ T cells in the BLNs also suggests that the vaccine may contribute to the early control of *M. hyopneumoniae* infection. Similar observations of local CD8^+^ T cell activation have been reported in swine influenza virus studies in pigs [55, 61]. Interestingly, the presence of TNF-α^+^ CD3^−^CD4^−^CD8α^−^ cells, IL-17A-producing CD4^+^ T cells, and proliferating CD8α^+^ CTLs in the BLNs upon *M. hyopneumoniae* vaccination was shown for the first time. Studies on *M. hyopneumoniae* infection and/or vaccination have not yet investigated cellular immunity in local draining lymph nodes, but they provide strong evidence that the vaccine successfully induced cellular immunity in lymphoid tissues [62–64].

Following intramuscular vaccination, the combined vaccine rapidly induced systemic and local cellular immunity, characterized by increased IFN-γ^+^/TNF-α^+^ CD4^+^CD8α ^+^ T cells, NK cells, and proliferating T cells in peripheral blood. Vaccination also activated IL-17A-producing CD4^+^ T cells and cytotoxic CD8α^+^ T cells in bronchial lymph nodes, supporting early control of *M. hyopneumoniae* infection. Humoral and mucosal immunity were enhanced, with elevated specific antibodies in serum and BALF after challenge, reducing pathogen load. Furthermore, vaccinated piglets displayed lower pro-inflammatory cytokines (IL-1β, CXCL-8, TNF-α, and IL-17A) in BALF, alleviating excessive pulmonary inflammation. Although lung lesion scores were not significantly different, vaccinated animals showed milder clinical signs and reduced bacterial loads, with activation of innate immune cells further contributing to protective immunity against *M. hyopneumoniae*.

In conclusion, the combined vaccine triggered local and systemic humoral and cellular immune responses specific for *M. hyopneumoniae*. These vaccine-induced immune responses lead to reduced clinical signs and the DNA load of *M. hyopneumoniae* upon challenge infection. Together, our results show that the combined vaccine triggered *M. hyopneumoniae*-specific immune responses in pigs that protected piglets against clinical signs upon challenge infection with *M. hyopneumoniae*.

## Supplementary Information

**Additional file 1**. **Macroscopic and microscopic lung lesion scores at D35 in control and vaccinated pigs upon challenge infection.** C: not vaccinated but infected; V: vaccinated and infected. Pigs were vaccinated once with an inactivated *M. hyo* vaccine. (*n*) = number of animals in each group. MLCL: Macroscopic lung lesion score was determined on D35. MLL: Microscopic lung lesions were assessed on D35.

**Additional file 2**. **All parameters from the NC group. **Piglets (n = 45) were divided into 3 groups. 20 piglets were vaccinated on D0 (V) and challenged with *M. hyo* after 3 weeks (D21), 20 piglets were not vaccinated but challenged (C), the remaining 5 piglets were as the negative control (NC); Eight pigs (4 from the V and 4 from the C groups) were euthanized 2 weeks after challenge (D35), the remaining ones were euthanized at D49. Blood was collected on D0, D7, D21, D35 and D49. BALF was collected on D7, D35 and D49. Lung tissue and bronchial lymph nodes (BLN) were collected on necropsy day (D35 and D49). NC: not vaccinated and not infected. One Piglet (Number: 14) couldn’t collect the blood and BALF sample on D35. MLCL: Macroscopic lung lesion score was determined on D49. MLL: Microscopic lung lesions were assessed on D49. D0: vaccination; D21: experimental infection; D35: necropsy of 4 animals in each group; D49: necropsy of the remaining animals.

**Additional file 3.** **Correlations between the DNA load of M. hyopneumoniae, cytokines in BALF or %IgA area in lung tissue and clinical signs (RDS, MLCL and MLL) in the C or V group.** The figure only shows the data with significant correlations; for the other parameters, the correlations were not statistically different (*P *> 0.05). The blue dots represent the data from the control group (C), and the light green dots represent the data from the vaccinated group (V). RDS: Respiratory Disease Score (RDS) was assessed daily. MLCL: Macroscopic lung lesion score was determined on D49. MLL: Microscopic lung lesions were assessed on D49.

**Additional file 4**. **Gating strategy to assess cytokine production and proliferation by T cells with CytExpert software.** All plots shown are obtained from *M. hyopneumoniae*-stimulated cells, isolated from piglets of the control (C) and vaccine (V) groups.

**Additional file 5**. **Representative dot plots of cytokine production in blood by T cell subsets with CytExpert software.** All plots shown are obtained from *M. hyopneumoniae*-stimulated T cells, isolated from piglets of the control (C) and vaccine (V) groups with three conditions (Medium, Concanavalin A and *M. hyopneumoniae*).

**Additional file 6**. **Representative dot plots of cytokine production in Bronchial lymph nodes by T cell subsets with CytExpert software.** The %CD3-CD4-CD8α- TNF-α+ producing dot plots shown T cells isolated from piglets in the C and V groups under three conditions (Medium, Concanavalin A, and *M. hyopneumoniae*) on D49.

**Additional file 7**. **Representative dot plots of T cell proliferation in blood.** The %CD3+ proliferation dot plots shown are obtained from T cells, isolated from piglets of the C and V groups with three conditions (Medium, Concanavalin A and *M. hyopneumoniae*).

## Data Availability

No datasets were generated or analyzed during the current study.
